# Anodizing Tungsten Foil with Ionic Liquids for Enhanced Photoelectrochemical Applications

**DOI:** 10.3390/ma17061243

**Published:** 2024-03-08

**Authors:** Elianny Da Silva, Ginebra Sánchez-García, Alberto Pérez-Calvo, Ramón M. Fernández-Domene, Benjamin Solsona, Rita Sánchez-Tovar

**Affiliations:** IQCATAL Group (Heterogeneous Catalysis), Chemical Engineering Department (ETSE), Universitat de València, Av. Universitat s/n, 46100 Burjassot-Valencia, Spain; elianny.silva@uv.es (E.D.S.); ginebra.sanchez@uv.es (G.S.-G.); alberto.perez-calvo@uv.es (A.P.-C.); ramon.fernandez@uv.es (R.M.F.-D.)

**Keywords:** ionic liquid, organic dye degradation, tungsten oxide, photoelectrocatalysis, water splitting

## Abstract

This research examines the influence of adding a commercial ionic liquid to the electrolyte during the electrochemical anodization of tungsten for the fabrication of WO_3_ nanostructures for photoelectrochemical applications. An aqueous electrolyte composed of 1.5 M methanesulfonic acid and 5% *v*/*v* [BMIM][BF_4_] or [EMIM][BF_4_] was used. A nanostructure synthesized in an ionic-liquid-free electrolyte was taken as a reference. Morphological and structural studies of the nanostructures were performed via field emission scanning electron microscopy and X-ray diffraction analyses. Electrochemical characterization was carried out using electrochemical impedance spectroscopy and a Mott–Schottky analysis. From the results, it is highlighted that, by adding either of the two ionic liquids to the electrolyte, well-defined WO_3_ nanoplates with improved morphological, structural, and electrochemical properties are obtained compared to samples synthesized without ionic liquid. In order to evaluate their photoelectrocatalytic performance, the samples were used as photocatalysts to generate hydrogen by splitting water molecules and in the photoelectrochemical degradation of methyl red dye. In both applications, the nanostructures synthesized with the addition of either of the ionic liquids showed a better performance. These findings confirm the suitability of ionic liquids, such as [BMIM][BF_4_] and [EMIM][BF_4_], for the synthesis of highly efficient photoelectrocatalysts via electrochemical anodization.

## 1. Introduction

In the last two decades, due to the advantages of nanostructured materials, such as their large surface area in relation to their volume and their high activity, they have been incorporated into a wide variety of energy applications, such as the production of hydrogen from water molecules, solar panels, etc., as well as environmental applications such as wastewater treatment, treatment of air effluents, and so on [1,2,3]. Recently, the use of ionic liquids (ILs) in the synthesis of nanostructured catalysts has become widespread, since they offer very attractive alternatives to traditional liquid organic solvents and solid salts. This is a result of the suitable physicochemical characteristics of these ILs, which include a greater conductivity, a low vapor pressure, nonflammability, and good thermal and chemical stability [4,5,6], in addition to their selective combination of cations or anions depending on the subsequent application and their treatability and reusability, greatly improving their environmental impacts and contributing to sustainability [5,6]. 

Electrochemical anodization is one of the many methods for synthesizing nanostructures from semiconductor metal oxides. It offers sufficient scalability and comparatively easy operation, giving excellent control over the morphology of the nanostructures and their physicochemical properties [7]. Anodization is carried out at moderate temperatures and atmospheric pressure, and the morphology and dimensions of the nanostructures can be altered by varying the number of operation parameters, including the temperature, electrolyte composition, and applied cell potential. This makes anodization a very suitable technique with a minimal environmental impact [8]. Some other methods have been studied to create WO_3_ nanostructures with a wide variety of morphologies (nanowires, nanopores, nanoflakes, nanoplates, etc.) such as hydrothermal methods [9], solvothermal methods [10], deposition processes (laser deposition [11], sol–gel [12], electrodeposition [13,14], chemical vapor deposition [15], RF sputtering [16,17], spin-coating [18]), and combustion. However, they use corrosive reactants and large amounts of energy and they are more complex to operate in comparison with anodization techniques.

Numerous semiconductor metal oxides have been studied by scientists for a variety of environmental and energy applications due to their photocatalytic activity [19,20]. By shining light on a nanostructured photocatalyst and applying a potential at the same time, matter such as dyes can be degraded. In this process, a positively charged hole is simultaneously created in the valence band and an excited electron in the valence band moves into the conduction band. They migrate to the surface of the photocatalyst, where they participate in the redox reaction of water and produce OH^−^ radicals, which are responsible for oxidizing the organic species present [21,22]. In the same way, during photoelectrochemical water splitting, electron–hole pairs are photogenerated in the conduction and valence layers, respectively. Then, they separate, and holes oxidize the water molecules to create O_2_ and H^+^ on the surface of the semiconductor, and electrons in the counter electrode reduce H^+^ into gaseous hydrogen [23,24,25].

Most of the many investigations that have been conducted with commercial ILs as solvents in the creation of nanostructured catalysts are centered on the fabrication of TiO_2_ nanostructures. The first investigation into ionic-liquid-based titanium anodization was reported by Schmuki et al. They showed that self-organized layers of TiO_2_ NTs may be produced directly on a titanium surface using anodization in 1-n-butyl-3-methyl-imidazolium tetrafluoroborate, [BMIN][BF_4_] [26]. Wender and colleagues subsequently synthesized titanium dioxide nanostructures with the same IL to evaluate them as photocatalysts for methyl orange photodegradation and hydrogen evolution from water/methanol mixtures [27]. Other studies have also shown improvements in the behavior of anodized titanium foils using this ionic liquid and some others like 1-ethyl-3 methylimidazolium tetrafluoroborate, [EMIN][BF_4_]; 1-butyl-3-methyl-imidazolium chloride, [BMIM][Cl]; 3-methyl-1-octyimidazolium tetrafluoroborate, [OMIM][BF_4_]; and 1-butylpiridinium chloride, [BPy][Cl] [28,29,30]. 

However, these different compounds have been rarely employed as solvents during electrochemical anodization on other types of semiconductor metal oxides and they could greatly contribute to enhancing their photoelectrochemical performance. Considering this, tungsten oxide (WO_3_) is drawing a lot of attention because of its natural abundance, sufficient conductivity, resistance to photocorrosion, low band gap value (between 2.5 and 3.0 eV [31,32,33]), and large hole diffusion (150 nm) [34]. Furthermore, the chemistry of tungsten and the ability of various compounds (ligands) to generate tungsten complexes and alter the electrolyte composition during anodization provide the opportunity to optimize the size and morphology of nanostructures [35]. In this regard, ILs may be employed as complexing agents for tungsten during the anodization process to produce WO_3_ nanostructures with different morphologies and sizes. The ionic liquids [BMIN][BF_4_] and [EMIM][BF_4_] contain fluoride species [F^−^] that act as monodentate ligands which form stronger bonds (higher stability) with tungsten, increasing the dissolution rate of the WO_3_ layer, which will then precipitate on the surface. They also delay the precipitation of tungstic acids on the electrode surface, forming nanostructures with better behavior [36]. In addition, the organic part of these molecules is short, which could facilitate interactions between the inorganic part [BF_4_]^−^ and the electrolyte and substrate. 

However, not many studies report the use of these materials in the synthesis of tungsten oxide nanostructures. N-methyl-pyrrolidinium tetrafluoroborate, a protic ionic liquid (PIL), was used by Go et al. to synthesize WO_3_ nanoplates, and their findings demonstrated an improvement in all electrochromic parameters when compared to an acid medium electrolyte without ILs [18]. 

Likewise, within the different applications mentioned, and in order to evaluate the photoelectrocatalytic performance of the WO_3_ nanostructures synthesized with the commercial ionic liquids [BMIN][BF_4_] and [EMIN][BF_4_], they will be used to carry out the splitting of water molecules for hydrogen production (with implications for energy applications) and the decomposition of the methyl red dye (with implications for the environment). Methyl red (MR) is an anionic organic dye that is produced as waste in the course of many different industries operations, including paper, pulp, plastic, leather, and textile industries, and so forth [37]. Various physicochemical and biological approaches, including chemical oxidation, ion exchange, and biodegradation, have been employed over the years to remove this pollutant from wastewater because of its high toxicity and health concerns to humans. Nevertheless, all of them have certain drawbacks, some of which include producing a lot of extra waste or their very high costs [2,37]. However, photoelectrocatalytic decomposition is promoted as a quick and easy method for removing this kind of organic contaminant. 

The primary goal of this work is to conduct a comprehensive analysis of the physicochemical and electrochemical properties of WO_3_ nanostructures anodized with the commercial ILs [BMIN][BF_4_] and [EMIN][BF_4_], ionic liquids which have not been traditionally used for this purpose. Furthermore, this investigation uses the nanostructures with the promising properties provided by ILs in applications in which they have not been tested before, such as the photoelectrochemical splitting of water and in the photodegradation of methyl red.

## 2. Materials and Methods

### 2.1. Synthesis by Electrochemical Anodization

Tungsten oxide nanostructures were synthesized by electrochemical anodization at 50 °C. A tungsten foil (with 1.32 cm^2^ exposed to the electrolyte) was used, and a potential difference between the tungsten sheet and the cathode (platinum foil) of 20 V for 4 h was applied. The anodization system was composed of a vertical cell consisting of a single compartment where the area exposed to the electrolyte was controlled by an O-ring. The synthesis of the nanostructures was carried out in an aqueous electrolyte with 1.5 M methanesulfonic acid and 5% *v*/*v* of [BMIM][BF_4_] or [EMIM][BF_4_] (labeled as BMIM and EMIM, respectively). To compare the obtained results, an ionic-liquid-free electrolyte was utilized (identified as Blank). After, the samples were rinsed with water, dried with a nitrogen stream, and annealed at 600 °C for 4 h.

### 2.2. Morphological and Structural Characterization of the Nanostructures (FESEM and XRD)

Morphological characterization of the nanostructures was performed using a field emission scanning electron microscope (FESEM) Hitachi S4800, at an accelerating potential of 5 kV. Using this technique, images were taken of the top of the nanostructures and of the cross-section (scratching the surface of the samples).

Additionally, the samples were subjected to X-ray diffraction (XRD) analysis, making use of a Bruker D8AVANCE diffractometer (Bruker, Billerica, MA, USA) fitted with a Cu Kα1 monochromatic source.

### 2.3. Electrochemical Characterization

Electrochemical characterization was performed via electrochemical impedance spectroscopy (EIS). A three-electrode single compartment cell was used: the nanostructure was the working electrode (with an exposed area of 0.5 cm^2^), a platinum tip was the counter electrode and an Ag/AgCl electrode was the reference electrode. This test was carried out in 0.1 M H_2_SO_4_ as the electrolyte. Using a potentiostat (PalmSens4, PalmSens, Houten, The Netherlands) at a constant potential of 1 V_Ag/AgCl_, a frequency scan was applied from 10 kHz to 10 mHz with an amplitude of 10 mV. Furthermore, in a similar experimental setup, a Mott–Schottky analysis was performed, scanning the potential from 1 to 0 V_Ag/AgCl_ with a frequency of 5000 Hz.

### 2.4. Application of WO_3_ Nanostructures

#### 2.4.1. Photoelectrochemical Production of Hydrogen

The tungsten oxide nanostructures were used to produce hydrogen from the photoelectrochemical splitting of water. These tests were carried out in a three-electrode single compartment cell using the samples as a working electrode, a platinum tip as a counter electrode, and an Ag/AgCl reference electrode. The samples were masked with an O-ring in the cell, exposing 0.5 cm^2^ of each one to the 0.1 M H_2_SO_4_ electrolyte. During this analysis, the surface of the WO_3_ nanostructures was illuminated with a UV light (λ = 365 nm, 100 mW·cm^−2^) while scanning the potential from 0 to 1.04 V_Ag/AgCl_ at a rate of 0.005 V/s (using a PalmSens4 potentiostat). Then, the same nanostructure was scanned in the same potential range, but this time in the dark.

#### 2.4.2. Photoelectrodegradation of Methyl Red

The application of the samples as photoelectrocatalysts in the photoelectrochemical degradation of methyl red dye in sulfuric acid 0.1 M was tested. A glass cell with a quartz window and three electrodes immersed in the dye solution was used for this analysis. The nanostructure was used as a working electrode (with an exposed area of 1.32 cm^2^), a platinum tip was used as a counter electrode and an Ag/AgCl electrode was used as a reference. The nanostructure was illuminated with a UV lamp (λ = 365 nm, 100 mW·cm^−2^), and with a potentiostat (PalmSens4), a potential of 1 V was applied. 

**Figure 1:**
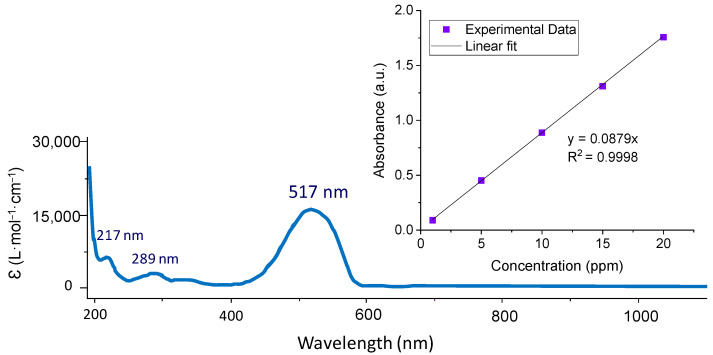
UV absorbance spectra of methyl red (inset: relationship obtained between absorbance “y” and methyl red concentration “x” at a wavelength of 517 nm).

Dye degradation was monitored every 10 min for 1 h using a quartz cell in a spectrophotometer (Cecil CE 1011, Cecil Instruments Limited, Cambridge, UK) at a wavelength of 517 nm. Figure 1 displays the UV absorbance spectra of the methyl red solution; the maximum absorption peak is in accordance with the literature [38]. The calibration of absorbance vs. concentration obtained at 517 nm is shown in the inset in Figure 1.

To check the accuracy of the method, the % recovery of three series of three samples with different concentrations (1, 5, 10 ppm) of methyl red has been calculated, showing that the method has a recovery percentage between 97.84 and 99.85. The precision of the method was also determined using three samples with different concentrations (1, 5, 10 ppm) of methyl red and analyzing each one three times and calculating the relative standard deviation (RSD). The results show that the method precisely obtains values below 1.8% RSD.

## 3. Results and Discussion

### 3.1. Synthesis by Electrochemical Anodization

**Figure 2:**
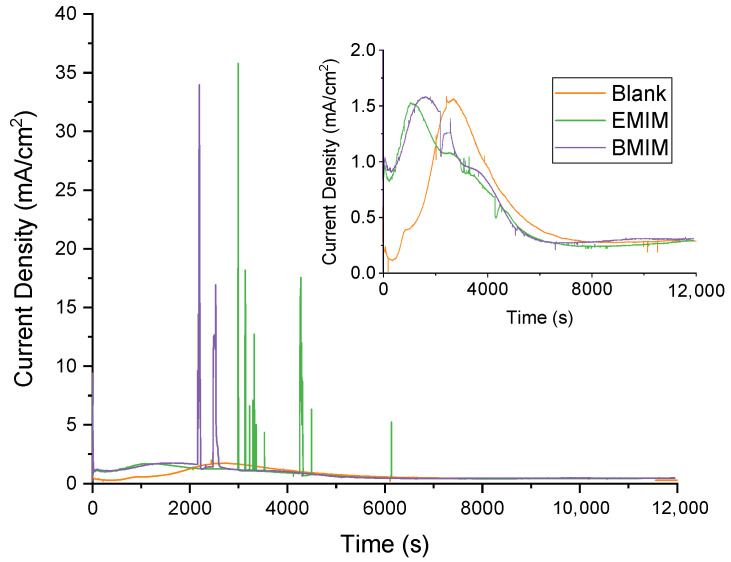
Current density curves recorded during electrochemical anodization at 20 V and 50 °C in different electrolytes (with and without IL).

It is important to mention that different concentrations (5%, 10%, 30%) of [BMIM][BF_4_] and [EMIM][BF_4_] were used to anodize tungsten. However, the best photoelectrocatalytic performance (Figure S1) was achieved by low-IL-concentration nanostructures (5%). Figure 2 displays the current density–time data registered during anodization in the blank electrolyte (without ILs) and in both ionic liquids (EMIM and BMIM). A magnified inset plot is shown to enable a proper view the current density transients of each sample. In this figure, the current density values reached during the synthesis process and their shapes can be examined. 

The blank electrolyte sample exhibits the shape of a typical dissolution–precipitation formation mechanism [39,40]. First, the current density steadily drops due to the growth of a compact WO_3_ layer; then, the high concentration of protons in the solution causes the dissolution of this layer and a resulting current density increase (second stage). Finally, the current density drops once more when the soluble tungsten species (created immediately before) reach supersaturation levels and precipitate as tungstic acid (hydrated and amorphous WO_3_·2H_2_O) in the form of nanostructures on the sample. Equations (1)–(3) represent these three processes that take place during anodization:

(1) $$W+3H_{2}O\to {WO}_{3}+{6H}^{+}+{6e}^{-}$$

(2) $${WO}_{3}+{2H}^{+}\to {{WO}_{2}}^{2+}+H_{2}O$$

(3) $${{WO}_{2}}^{2+}+{3H}_{2}O\to {WO}_{3}\cdot {2H}_{2}O+{2H}^{+}$$

Some differences can be elucidated when 5% of either ionic liquid was added to the electrolyte, for instance, in the moment the current density reaches its maximum. This maximum is reached sooner in the presence of 5% IL due to the lower dielectric constants of the ionic liquids and, therefore, of the anodizing electrolyte containing them. A lower dielectric constant facilitates the precipitation of the soluble tungsten species generated in stage two of the anodization process. Therefore, it is reasonable that the current density peak belonging to this part of the synthesis is reached earlier in electrolytes with lower dielectric constants (EMIM or BMIM). Moreover, this behavior is reflected in the total charge of the anodization process of the samples (Table 1). The addition of either of the ionic liquids to the electrolyte accelerates the dissolution–precipitation mechanism associated with the formation of WO_3_ nanostructures and, therefore, the total charge density is higher.

On the other hand, in the current transients recorded during anodization for the samples synthesized with EMIM or BMIM, diverse peaks can be appreciated, which correspond to the formation of pitting or the localized dissolution of tungsten due to the presence of BF^−^ ions that tend to form soluble tungsten complexes [41,42]. This result has been observed before when using other fluoride-containing electrolytes. As has been outlined before, with NaF, fluoride ions formed strong bonds with tungsten since they act as monodentate ligands. Then, due to the acidic electrolyte, fluoride ions encouraged localized dissolution of the formed WO_3_ layer and developed soluble fluoride complexes [36]. Specifically, methanesulfonic acid can excellently solubilize metal salts and it is an environmentally friendly electrolyte [43].

Furthermore, comparing both IL curves, it can be noted how stage two occurs earlier for the EMIM electrolyte than for the BMIM electrolyte. This can be attributed to the organic contribution, which is the result of larger organic molecules in the electrolyte. This leads to steric hindrance in the interaction between the oxide layer and fluoride ions.

### 3.2. Morphological and Structural Characterization of the Nanostructures (FESEM and XRD)

**Figure 3:**
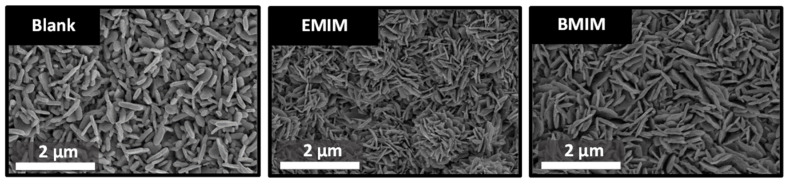
FESEM images of tungsten oxide nanostructures synthesized via electrochemical anodization with different electrolyte solutions (with and without IL).

The FESEM images shown in Figure 3 were taken to study the influence of ionic liquids on the WO_3_ nanostructures synthesized via electrochemical anodization. The images show the morphology of the samples synthesized without ionic liquid and of samples for which 5% *v*/*v* EMIM or BMIM was added to the anodization electrolyte. In the three studied cases, the formation of nanoplates can be observed, which increase in length and number with the addition of ionic liquid. This behavior is in agreement with what was expected according to the anodization curves studied in the previous section, since the nanoplates synthesized with EMIM or BMIM begin to precipitate before those formed with the free electrolyte. According to Figure 2, the second stage (precipitation stage) starts at 1100 or 1700 s for the samples synthesized with EMIM or BMIM, respectively, while, for the blank electrolyte, it starts at ~2000 s. Therefore, the nanoplates synthesized with ionic liquid have more time to form and grow. All of this leads to a higher surface area of the nanostructures. Moreover, the larger number of nanoplates in the EMIM sample can also be explained by the precipitation stage beginning earlier, as the peak is reached before for this nanostructure (Figure 2). 

Table 2 shows three parameters related to WO_3_ nanostructures: nanoplate length, nanoplate thickness and WO_3_ layer thickness. These measurements were determined from the top view and cross-sectional images of the nanostructures taken via FESEM (see Supporting Information, Figure S2). The results indicate that the nanoplates obtained by adding EMIM or BMIM to the anodization electrolyte are longer and thinner. In addition, the thickness of the WO_3_ layer increases with the presence of ionic liquids. This behavior is consistent with what has been previously mentioned regarding the earlier precipitation of tungsten species during anodization. The BF^−^ ions from ionic liquids tend to form soluble tungsten complexes and, as the anodization process continues, the supersaturation condition is reached faster, so that soluble species start to precipitate on the sample surface and favor the formation of thicker layers.

**Figure 4:**
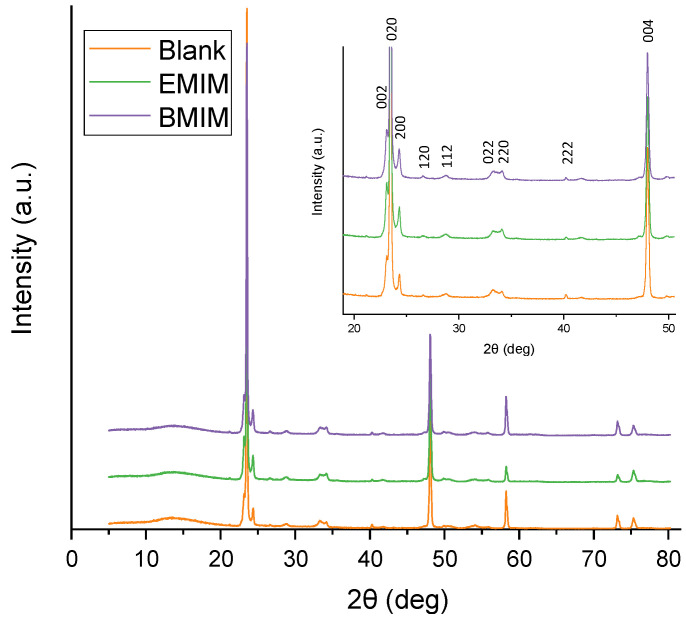
X-ray diffraction patterns for WO_3_ nanostructures after anodization in different electrolytes (with and without IL) and magnification of the different monoclinic phase peaks.

Figure 4 shows the X-ray diffractogram of WO_3_ nanostructures synthesized by electrochemical anodization after being calcined at 600 °C for 4 h. At this annealing temperature (600 °C), the WO_3_ nanostructures show a high crystalline structure, a lower resistance to charge transfer, and a higher dopant density, leading to a better photoelectrochemical performance [44].

As can be seen, the three samples present the characteristic peaks of monoclinic tungsten (JCPDS no. 43-1035). This result agrees with previous studies, as other researchers have reported that after annealing, amorphous WO_3_ turned into a crystalline monoclinic phase [45,46,47]. Therefore, it is clear that the addition of ionic liquid in the electrolyte does not affect the crystalline properties of the synthesized nanostructures. Using Scherrer’s Equation (4), the size of the crystallites has been determined (Table 3).

(4) $$D=\frac{\kappa \lambda }{FWHM\cdot cos(\theta )}$$

where D is the crystallite size (nm), κ is the shape factor (0.9), λ is the X-ray wavelength (0.1542 nm), FWHM is the width at half maximum intensity (rad), and θ is the Bragg angle [48]. In this case, the triplet of peaks appearing at 23.15, 23.48, and 24.25°, corresponding to the crystallographic planes (002), (020), and (200) and which can be assigned to the main monoclinic WO_3_ peaks (JCPDS no. 43-1035), have been used. The rest of the peaks also belong to the monoclinic WO_3_ phase, which has been described as the most stable one [49,50].

The obtained results indicate that the addition of ionic liquid in the electrolyte causes a decrease in the crystallite size. This behavior is due to the presence of [BF_4_]^−^ ions in the electrolyte since they favor the formation of tungsten complexes and influence the dissolution rate of WO_3_. As a consequence, the surface area of the nanostructures increases, leading to the decrease in the crystallite size of the samples. This result is favorable for the photocatalytic performance of the nanostructures, since for samples with smaller crystallite sizes, the transfer of photogenerated charge carrier pairs is enhanced and their recombination probability is reduced [51,52].

### 3.3. Electrochemical Characterization

**Figure 5:**
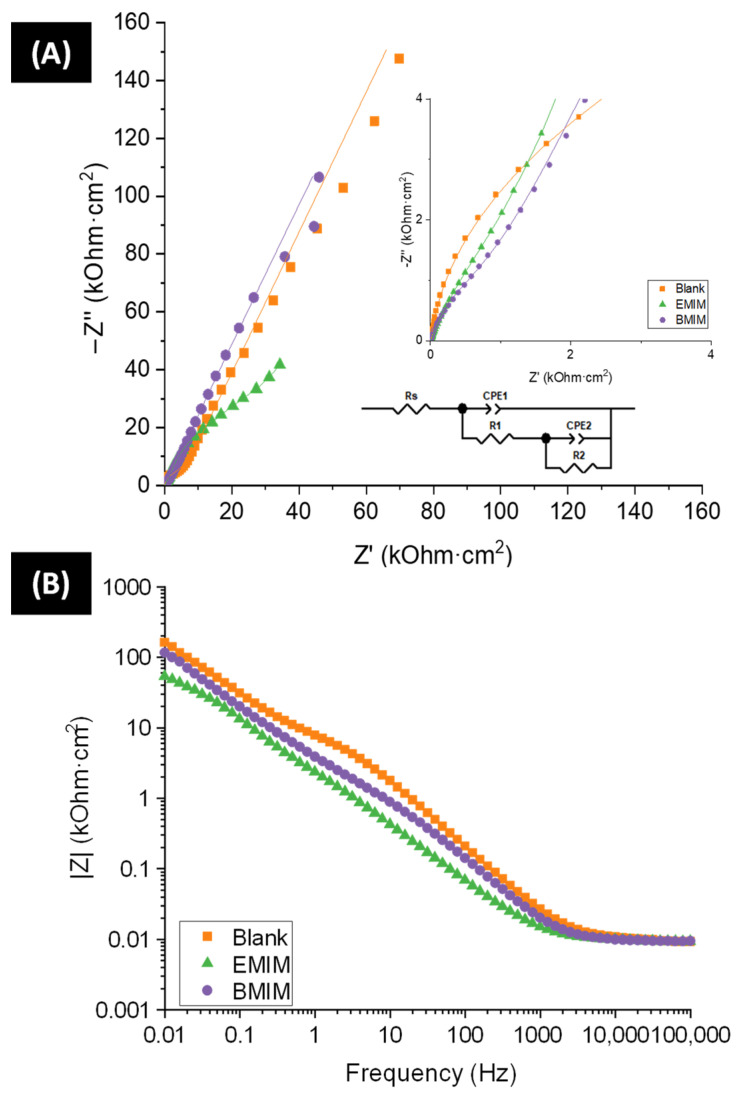
(**A**) Nyquist plots of WO_3_ nanostructures formed in electrolytes with and without IL (continuous line represents the fitting to the equivalent circuit), (**B**) Bode module plot of WO_3_ nanostructures formed in electrolytes with and without IL.

Electrochemical impedance spectroscopy tests were performed to learn about the resistance to charge transfer processes in each photoelectrode. Figure 5 shows the Nyquist (A) and Bode module (B) data for the WO_3_ nanostructures in the frequency range of 100 kHz to 1 mHz obtained at a potential of 1 V versus Ag/AgCl 3 M KCl (Bode phase plots in Figure S3).

Regardless of the sample, every Nyquist plot presents two semicircles, each one corresponding to one of the regions of the Bode module plots with different slopes. First, the charge transfer response of the oxide/electrolyte interface can be linked to the semicircle obtained at high and intermediate frequencies (see inset of Figure 5A), which can reveal information about the active surface area of the catalysts. The one at low frequencies is usually related to the compact layer of oxide formed under the nanostructure [53,54].

Generally, the semicircle amplitude is proportional to the impedance of the related electrochemical process. Observing Figure 5, the Blank sample presents the largest semicircle. This fact is confirmed in Figure 5B, where the values registered at the lowest frequency in the plot represent the total resistance offered by each nanostructure (R_T_). These results are presented in Table 4. As observed, the Blank sample presents a higher R_T_. It is confirmed that the addition of ionic liquid to the anodizing electrolyte decreases the total resistance to charge transfer of the tungsten nanostructures. It is important to highlight that the total resistance (R_T_) is lower for the WO_3_ nanostructure synthesized with EMIM than for the one synthesized with BMIM. This result is due to the shorter structural chain of EMIM which, therefore, makes it a slightly better complexing agent than BMIM. Additionally, EMIM nanostructures with larger surface areas have been obtained and this decreases the resistance they offer and increases their donor density [55].

To quantitatively analyze the EIS results, an electrical equivalent circuit with two parallel R-C time constants was used, as illustrated in Figure 5A (depicted in more detail in Figure S4). In this circuit, the non-ideality of the system has been considered by using constant phase elements (CPEs) as a substitute for pure capacitors [56,57]. An impedance fitting analysis was performed with the software ZView4, following the equation shown below (5):

(5) $$Z=R_{S}+\frac{R_{1}+R_{2}+R_{1}R_{2}Y_{2}{\left(j\omega \right)}^{\alpha_{2}}}{1+R_{1}Y_{1}{\left(j\omega \right)}^{\alpha_{1}}+R_{2}Y_{2}{\left(j\omega \right)}^{\alpha_{2}}+R_{2}Y_{1}{\left(j\omega \right)}^{\alpha_{1}}+R_{1}R_{2}Y_{1}Y_{2}{\left(j\omega \right)}^{\alpha_{1}}{\left(j\omega \right)}^{\alpha_{2}}}$$

From this fitting, the charge transfer resistance in the active parts of the nanostructure/electrolyte interface (R_1_) can be quantified. The results are displayed in Table 4, taking into consideration that, for all cases, the chi-squared values were lower than 10^−3^, validating the circuit used. The rest of the parameters in Equation (5) appear in Table S1. As expected, R_2_ values (bulk section) are extremely high as they belong to the compact oxide layer beneath the active part of the nanocatalysts. Therefore, these values are not considered at any point, since this part of the samples does not take part in the studied reactions (only the nanostructured top layers take part).

As expected, charge transfer in the active part of the WO_3_ nanostructures is also improved by the addition of ionic liquid during the anodization process, which can be related to the higher surface area of the IL samples, as described before. Generally, this effect would be beneficial for the photoelectrochemical performance of these catalysts since a low resistance improves electron transfer and hole diffusion, leading to a better photoresponse [58,59,60].

**Figure 6:**
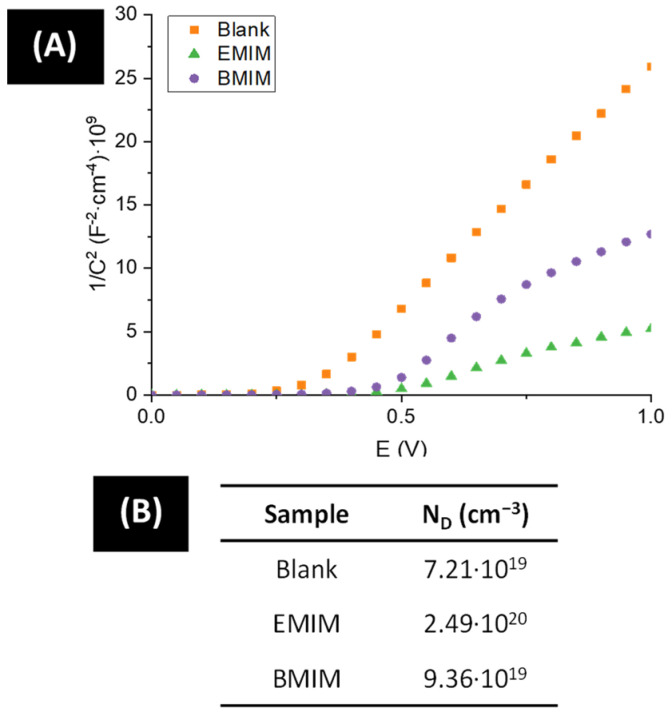
(**A**) Mott–Schottky plots obtained at a frequency of 5 kHz for WO_3_ nanostructures anodized with varied ILs. (**B**) Donor density (N_D_) calculated from MS plots for the nanostructures synthesized with different ILs.

These findings are in agreement with what the Mott–Schottky plots show in Figure 6A. The synthesized tungsten oxide nanostructures are n-type semiconductors, as the positive slopes of the plots reveal. Therefore, electrons are the dominant charge carriers, and with the use of the Mott–Schottky Equation (6), it is possible to calculate the donor density (N_D_) of each sample:

(6) $$\frac{1}{C^{2}}=\frac{1}{{C_{H}}^{2}}+\frac{2}{e\cdot \varepsilon_{T}\cdot \varepsilon_{0}{\cdot N}_{D}}\cdot \left(E-E_{FB}-\frac{k\cdot T}{e}\right)$$

where *C_SC_* is the space charge layer capacitance, *C_H_* is the Helmholtz layer capacitance, *e* the electron charge (1.60 × 10^−19^ C), ε_0_ is the vacuum permittivity (8.85 × 10^−14^ F/cm), ε is the dielectric constant of WO_3_ (50 [61,62,63]), *E* is the applied potential, *k* is the Boltzmann constant (1.38 × 10^−23^ J/K), and *T* is the absolute temperature [64]. The values of N_D_ for each nanostructure are shown in Figure 6B.

For tungsten oxide, the donor density is linked to oxygen vacancies, since these are the dominant defects in these nanostructures. From Figure 6B, it can be inferred that the addition of ionic liquid in the anodization process increases the donor density of the samples. A higher number of oxygen vacancies has a positive impact on WO_3_ nanostructures when used as photoanodes, since the electrical conductivity increases and, therefore, their photoelectrochemical performance improves. However, oxygen vacancies can also act as electron traps, facilitating the recombination of photogenerated electron/hole pairs [65,66], which could lead to worse photoelectrochemical behavior of the nanostructures. 

### 3.4. Application of WO_3_ Nanostructures

#### 3.4.1. Photoelectrochemical Production of Hydrogen

**Figure 7:**
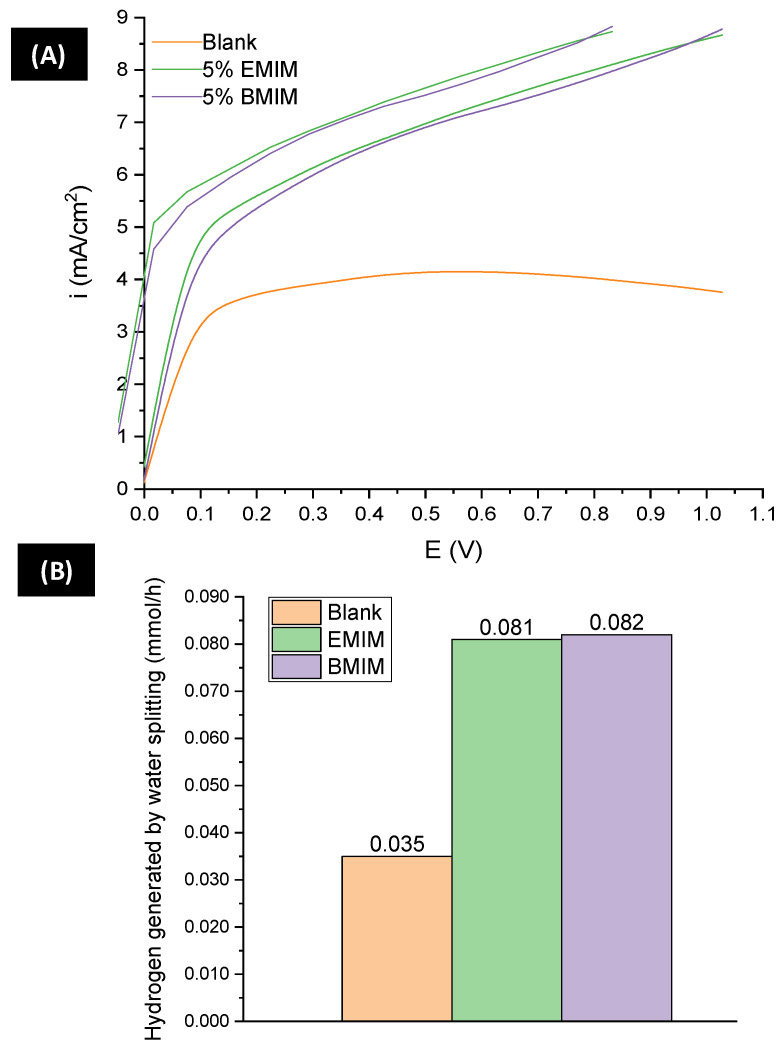
(**A**) Photocurrent transient vs. potential of tungsten oxide nanostructures synthesized by electrochemical anodization in different electrolyte solutions (with and without IL). (**B**) Number of moles of hydrogen generated during the splitting of water molecules.

Figure 7A shows the results obtained in the photoelectrochemical separation of water molecules after exposing the samples to illumination with a UV lamp and making a potential scan in the positive direction. According to what can be seen in the graph, the three samples present a good photoresponse, since an increase in the current density was recorded when illuminating the surface of the nanostructure. It is worth stating that when nanostructures are in the dark, the current density recorded is very close to 0 in all cases. Figure 7B exhibits the theoretical molar flow of hydrogen produced with the nanostructures at a potential of 1 V, calculated with Faraday’s law. According to these results, the nanostructures synthesized with ionic liquid exhibit a hydrogen production that is about 130% higher than that obtained with the blank sample. Furthermore, compared to what has been reported in the literature, these nanostructures exhibit a much higher hydrogen production than others. For example, other IL-synthesized nanostructures generate 81–82 µmol during water splitting, while CuCl/WO_3_ samples only generate 5 µmol after 30 min of exposure to UV light [67]. These results suggest that the proposed method of synthesis allows for obtaining efficient WO_3_ nanostructures with a better photoresponse, since their production of hydrogen is 16 times higher. In other study [68], tungsten oxide nanostructures were illuminated with UV light for water splitting applications, and thecurrent density reached maximum values of 0.5 mA/cm^2^ when applying 1V_Ag/AgCl_ using a light intensity of 50 mW/cm^2^. The current density values were more than 15 times lower than the ones achieved in this work at the same applied potential with 100 mW/cm^2^.

According to the morphological properties of the samples, it can be seen that the nanostructures synthesized with ionic liquid had a thicker nanostructured layer, an observation that favors the photoelectrocatalytic performance by increasing the surface area of the nanostructures [36,69].

Additionally, the three samples had a good crystalline structure; however, those synthesized with EMIM and BMIM presented a smaller crystallite size. Therefore, the probability of recombination of the electron/hole pair is lower than for the sample synthesized with the blank electrolyte [70]. 

Electrochemical characterization of the samples indicated that those that were anodized under the presence of ionic liquid had a considerably lower charge transfer resistance than the one synthesized with an ionic-liquid-free electrolyte. Therefore, it is implied that these nanostructures have better photoelectrocatalytic behavior, since electron transfer and hole diffusion are improved. A similar effect is obtained as a result of the number of defects present in the nanostructures, since other studies have shown that, with a higher number of vacancies, charge transfer processes are improved due to the better mobility of electrons [71,72]. 

#### 3.4.2. Photoelectrodegradation of Methyl Red

**Figure 8:**
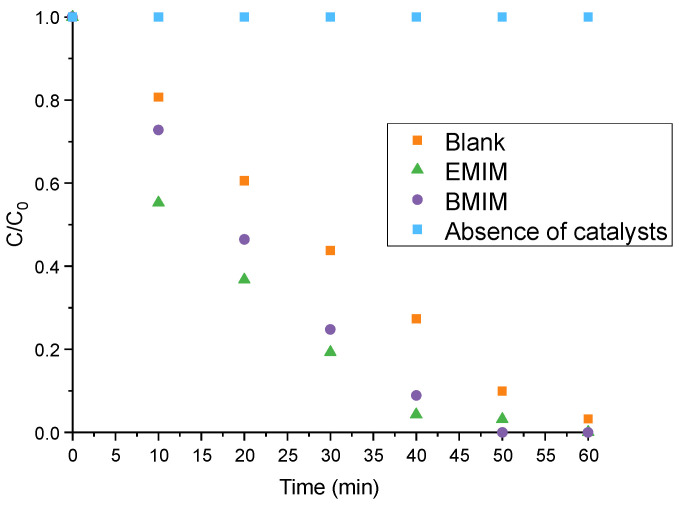
C/C_0_ of methyl red as a function of time during its photoelectrochemical degradation using a WO_3_ nanostructure synthesized with ionic liquid.

Tungsten oxide nanostructures were tested as photoelectrocatalysts in the degradation of methyl red dye in a 0.1 M sulfuric acid solution. The evolution of dye degradation over time can be seen in Figure 8.

In this analysis, a starting dye concentration of 20 ppm was used and, measuring the absorbance at ~517 nm using a spectrophotometer, the elimination of the dye was evaluated every 10 min. According to the obtained results, the concentration of methyl red without catalysts remained constant during the experiment, showing that there is no degradation of the dye. On the other hand, the nanostructures synthesized with ionic liquid exhibit a better performance than the sample synthesized with a blank electrolyte, since complete degradation of the dye was achieved after 60 or 50 min when using the nanostructures synthesized with EMIM or BMIN, respectively, while, for the reference sample, 60 min was not enough to degrade the whole dye. It is important to note that the sample anodized without ionic liquid always had a lower percentage of elimination than the anodized samples with ionic liquids. Taking a closer look at the degradation at 40 min, both nanostructures anodized with EMIM and BMIM achieve more than 90% MR degradation, while for the blank this value was close to 70%, which means that it is possible to reduce the energy consumption necessary to degrade a high percentage of methyl red dye using ILs as electrolytes. At 60 min, the sample without ILs managed to degrade most of the dye because it is also a nanostructured area with photoelectrocatalytic activity and, with longer durations, it has the capacity to completely degrade methyl red. 

In Figure S5, the degradation efficiency of each nanostructure is calculated. It can be appreciated how the EMIM nanostructure presents a better degradation percentage during most of the test, although the EMIM nanostructure requires 10 min more than the BMIM sample to achieve 100% degradation efficiency. These results are related to what has been mentioned above; that is, EMIM has a better complexing activity than BMIM, which leads to a greater dissolution of the oxide layer during the anodization and subsequently a greater precipitation with nanoplates of smaller sizes and with greater surface areas that benefit the photoelectrocalytic activity. Therefore, the use of EMIM seems more promising due to the obtained results. Despite this, Figure S5 reinforces the fact that both ionic liquids improve the photoelectrochemical behavior of the tungsten oxide nanostructures and both have a similar photoelectrocatalytic performance.

Considering the above-mentioned observations and the results of water splitting, the samples synthesized with EMIM or BMIM present a higher photoelectrocatalytic performance, since, as previously determined, their morphological, structural, and electrochemical properties were improved in relation to the sample synthesized with an ionic-liquid-free electrolyte [73]. In addition, the degradation efficiency obtained in this study is better than the 97% degradation of methyl red reached in 160 min with WO_3_ nanostructures under visible light illumination, but more favorable conditions were used in the latter example, such as a lamp of 500 W, an active surface area of 64 cm^2^, and a greater external potential of 1.5 V [74]. In similar degradation conditions but with another organic dye, methyl orange (similar structure), our nanostructure also provided better results [75]. Compared with other metal oxides such as TiO_2_ doped with Au, the WO_3_ nanostructures fabricated in the presence of EMIM and BMIM exhibit better degradation yields in less time, although in that study, two ultraviolet lamps were used during the degradation tests [76].

## 4. Conclusions

In this study, we proved that with the incorporation of commercial ionic liquids (EMIM or BMIM) to the electrolyte during electrochemical anodization of tungsten, it is possible to synthesize more efficient nanostructures for photoelectrochemical applications, such as photoelectrochemical hydrogen production and the degradation of methyl red dye.

To be more specific, with 5% *v*/*v* of either IL, the anodization process was faster, and this led to higher-surface-area nanostructures, since the nanoplate length increased from 0.48 µm (blank) to 0.60 µm (EMIM) and 0.64 µm (BMIM) and the layer thickness increased from 0.7 µm using the blank electrolyte to 1.5 µm and 1.2 µm when using EMIM and BMIM, respectively.

Furthermore, the formation of a WO_3_ monoclinic crystalline phase in the samples was improved by the addition of EMIM/BMIM, since the crystallite size decreased from 53.5 nm for the sample anodized in the blank electrolyte to 49.7 nm for the IL-anodized sample. 

Additionally, the use of ILs during electrochemical anodization of tungsten resulted in nanostructures with a higher number of oxygen vacancies, as the number rose from 7.21 × 10^19^ cm^−3^ to 2.46 × 10^20^ cm^−3^ and 9.36 × 10^19^ cm^−3^ for EMIM and BMIM samples, respectively. This led to a higher electrical conductivity that improved the photoelectrochemical performance.

In particular, during the splitting of water using EMIM and BMIM samples, the generation of hydrogen was increased by 132% and 135%, respectively, when compared to the hydrogen production of the blank sample. Moreover, with the application of IL samples as photoelectrocatalysts in the photoelectrodegradation of methyl red, a 100% degradation efficiency was achieved after 50/60 min. To sum up, these results exhibit how IL-anodized nanostructures can be used for efficient and fast methyl red degradation involving a lower energy consumption, since less time is needed for a higher percentage of elimination.

## Figures and Tables

**Table 1:** Total charge during electrochemical anodization of tungsten.

Sample	Total Charge (C)
Blank	9.5
EMIM	10.2
BMIM	10.8

**Table 2:** Morphological parameters of WO_3_ nanostructures.

Sample	Nanoplate Length (μm)	Nanoplate Thickness (μm)	WO_3_ Layer Thickness (μm)
Blank	0.48 ± 0.09	0.09 ± 0.02	0.7 ± 0.1
EMIM	0.60 ± 0.05	0.06 ± 0.01	1.5 ± 0.2
BMIM	0.64 ± 0.06	0.07 ± 0.01	1.2 ± 0.1

**Table 3:** Crystallite size determined via the Scherrer equation.

Sample	Crystallite Size (nm)
Blank	53.5
EMIM	49.7
BMIM	49.7

**Table 4:** Total (Bode module plots) and active part (equivalent circuit fitting results) resistances of the nanocatalysts.

Nanostructure	R_T_ (kOhm·cm^2^)	R_1_ (kOhm·cm^2^)
Blank	163.15	8.19
EMIM	53.95	1.38
BMIM	116.05	1.98

## Data Availability

Data are contained within the article and supplementary materials.

## Supplementary Materials

The following supporting information can be downloaded at: https://www.mdpi.com/article/10.3390/ma17061243/s1, Figure S1: Photocurrent transient vs. potential of WO_3_ nanostructures synthesized by electrochemical anodization in different electrolytes (with and without IL) and with different concentrations of BMIM and EMIM (5, 10 and 30%); Figure S2: FESEM images of the WO_3_ nanostructure synthesized by electrochemical anodization with blank electrolyte. Top view (left) and cross section (right); Figure S3: Bode-Module and Bode-phase plots WO_3_ nanostructures formed in electrolytes with and without IL; Figure S4: Equivalent circuit used for EIS fitting; Table S1: Results of EIS fitting analysis; Figure S5: Methyl red degradation efficiency of the different nanostructures.
