# Supplementary material for: Anodizing Tungsten Foil with Ionic Liquids for Enhanced Photoelectrochemical Applications
Source: Materials (Basel). 2024 Mar 8;17(6):1243. doi: 10.3390/ma17061243 (PMC10972159; doi:10.3390/ma17061243)
Supplement: Supplementary file 1 [file materials-17-01243-s001.zip › materials-2885918-supplementary.pdf]

Supporting Information

Anodizing tungsten foil with ionic liquids for enhancing photoelectrochemical applications

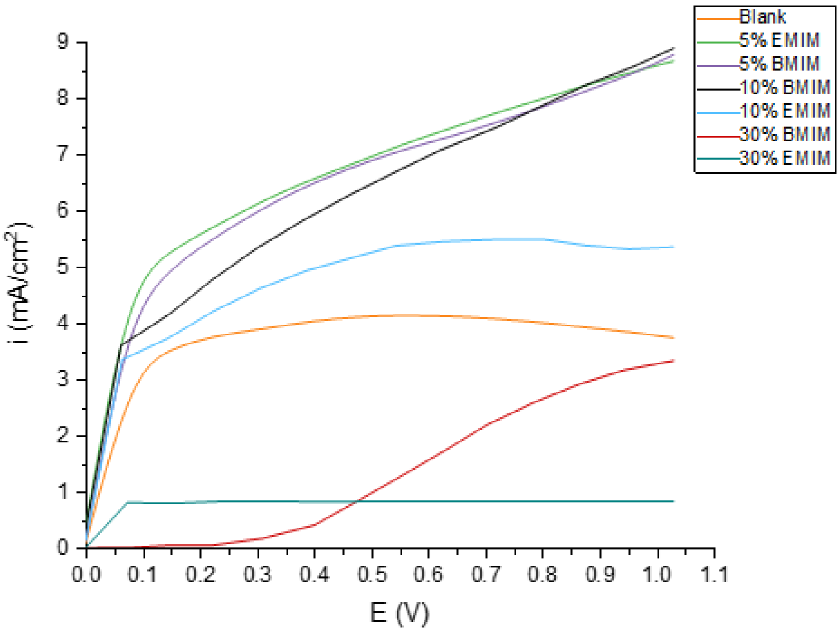

Figure S1. Photocurrent transient vs. potential of WO_3 nanostructures synthesized by electrochemical anodization in different electrolytes (with and without IL) and with different concentrations of BMIM and EMIM (5, 10 and 30%).

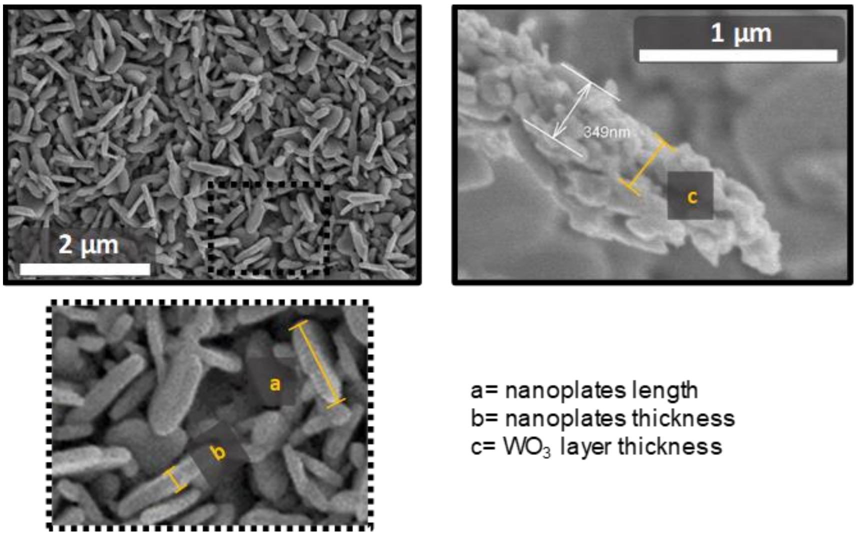

Figure S2. FESEM images of the WO_3 nanostructure synthesized by electrochemical anodization with blank electrolyte. Top view (left) and cross section (right).

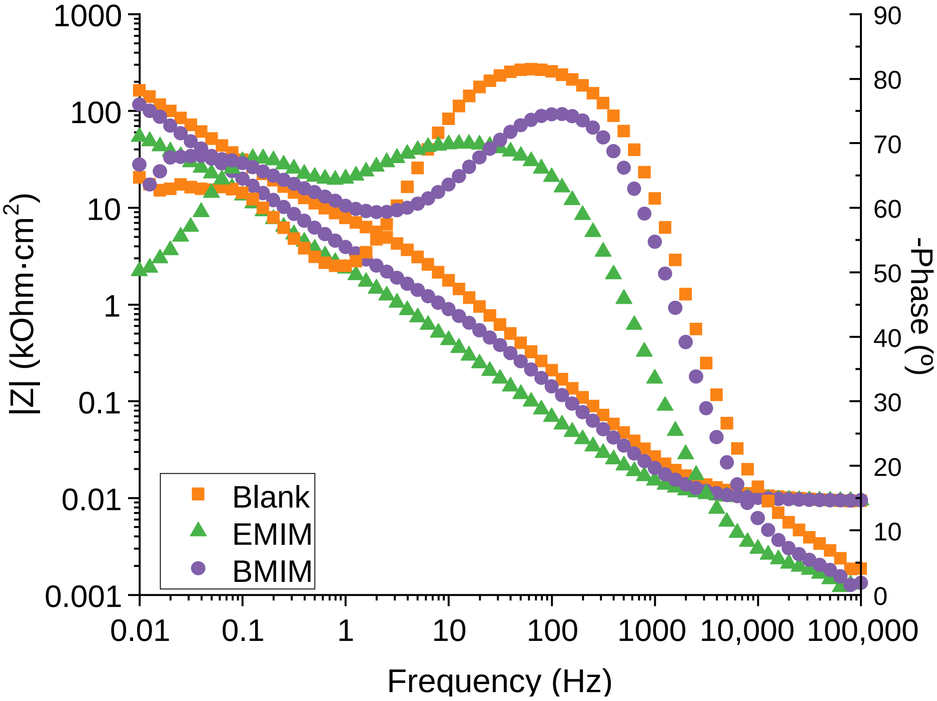

Figure S3. Bode-Module and Bode-phase plots WO_3 nanostructures formed in electrolytes with and without IL.

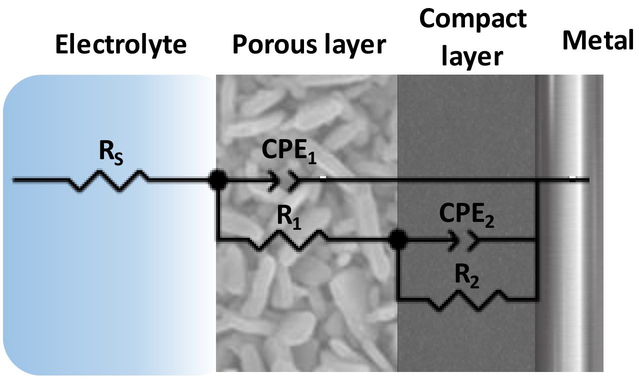

Figure S4. Equivalent circuit used for EIS fitting.

Table S1. Results of EIS fitting analysis

Nanostructure	R_S (Ohm)	CPE1-T $F\cdot s^{(\alpha-1)}/cm^2$	α_1	CPE2-T $F\cdot s^{(\alpha-1)}/cm^2$	α_2	R_2 (Ohm)
**Blank**	19.74	1.09E-05	0.9423	4.10E-05	0.7317	1E+20
**EMIM**	18.48	8.12E-05	0.8133	3.00E-05	0.3896	1E+20
**BMIM**	18.91	1.97E-05	0.9145	5.30E-05	0.7217	1E+20

Where: impedance values for CPE_i-T are calculated by $\frac{1}{Y_0\cdot(j\omega)^{\alpha}}$,

R_S is the resistance offered by the electrolyte

R_2 is the resistance of the bulk.

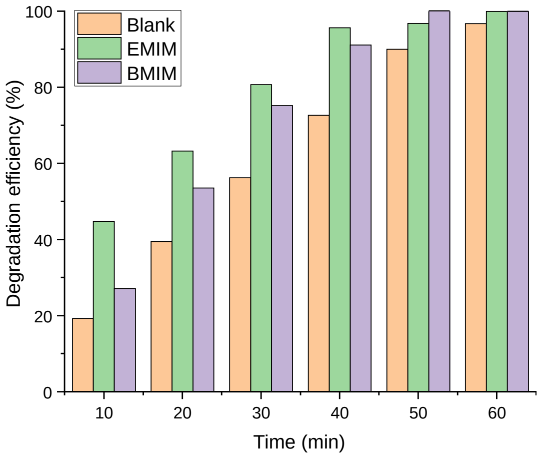

Figure S5. Methyl red degradation efficiency of the different nanostructures.
